# Stakes and expertise modulate conformity in economic choice

**DOI:** 10.1038/s41598-021-02793-z

**Published:** 2021-12-03

**Authors:** Jordanne Greenberg, Mimi Liljeholm

**Affiliations:** 1 Department of Cognitive Sciences, University of California, Irvine, Irvine, USA; 2 Center for the Neurobiology of Learning & Memory, University of California, Irvine, Irvine, USA; 3 Department of Cognitive Sciences, 2312 Social and Behavioral Sciences Gateway, University of California, Irvine, Irvine, CA 92697-5100 USA

**Keywords:** Psychology, Human behaviour

## Abstract

The influences of expertise and group size on an individual’s tendency to align with a majority opinion have been attributed to informational and normative conformity, respectively: Whereas the former refers to the treatment of others’ decisions as proxies for outcomes, the latter involves positive affect elicited by group membership. In this study, using a social gambling task, we pitted alignment with a high- vs. low-expertise majority against a hypothetical monetary reward, thus relating conformity to a broader literature on valuation and choice, and probed the countering influence of a high-expertise minority opinion. We found that the expertise of a countering minority group significantly modulated alignment with a low-expertise majority, but only if such alignment did *not* come at a cost. Conversely, participants’ knowledge of payoff probabilities predicted the degree of majority alignment *only* when a high-expertise majority endorsed a more costly option. Implications for the relative influences of expertise and stakes on conformity are discussed.

## Introduction

Ever since Solomon Asch^1^ demonstrated that individuals abandon their own superior judgements to align with an obviously incorrect but unanimous group of peers, the factors that influence conformity have been intensely studied across social sciences. Much of this work has focused on dissociating informational and normative conformity—while the former involves viewing others’ decisions as evidence, the latter is based on a desire to belong^2^—particularly with respect to the relative influences of majority size and expertise^3–7^. Notably, while alignment with a majority opinion might provide access to group resources, protect individuals from social rejection^8^, and even perpetuate charitable behavior^9^, it can also be maladaptive, as when degrading the “wisdom of the crowd”^10^, or inducing individuals to make suboptimal decisions^11^. In this study, we used a social gambling task to pit alignment with a high- vs. low-expertise majority against hypothetical monetary gain, and against the countering opinion of a high-expertise minority.

A substantial literature has addressed the relationship between perceived self-competence, group expertise and conformity^5–7^. For example, Costanzo et al.^5^ found that a subject made to believe, through experimenter-controlled feedback, that s/he was incompetent at judging the size of a set of stimuli was more likely to conform to a group with ostensibly high expertise than a group with low expertise. Similarly, in a study assessing the perceived nutritional value of fictitious dietary products, Lascu et al.^6^ found that participants’ self-assessed task competence modulated the influence of ostensible group expertise on conformity. The interaction between self-competence and group expertise has been interpreted as evidence for informational, rather than normative, conformity^12^. Of course, one might argue that perceived low self-competence leads to low self-esteem, thus enhancing the affirmational value of belonging. Moreover, membership in a group with greater expertise may confer greater perceived status and thus greater value. In general, in the absence of any stakes (e.g., real or hypothetical economic loss), it is difficult to discern how the subjective *value* of conforming contributes to decision making: Specifically, is the desire to belong strong enough to warrant an apparent decision cost?

Interestingly, Asch^1^ found that the presence of even a single dissenter, agreeing with the subject, substantially reduced the influence of the group, and similar results have been reported in more recent work on memory conformity^13^; it remains unclear, however, whether the expertise of the majority, and of the dissenting minority, modulates such effects. We predicted that, in the absence of stakes, minority expertise would be heavily weighed when majority expertise was low; conversely, if the majority endorsed an objectively incorrect (i.e., less rewarding) option, minority expertise would have a greater influence when majority expertise was high, since an incorrect *and* low-expertise majority should be dismissed regardless of the expertise of the minority. Finally, we expected that an individual’s objective competency—operationalized as the accuracy of reward probability estimates—would predict the tendency to align with high, but not with low, expertise.

## Methods

### Participants

Thirty-seven undergraduates at the University of California, Irvine (32 females, mean age = 21.2 $\pm$ 3.2) completed the study for course credit. Our primary hypothesis was that the influence of minority expertise on conformity would depend both on the expertise of the majority, and on whether majority alignment came at a cost. Using a small independent sample (n = 14), we calculated the three-way interaction score (i.e., the difference in conformity between high and low minority expertise, given high vs. low majority expertise, when conformity came at cost vs. when reward probabilities were equal), and obtained an effect size (partial eta squared; ${\eta }_{p}^{2}$) of 0.50. An a priori power analysis in G*Power (3.1.9.2;^14^) yielded a required sample size of 34 to achieve an 0.8 power to detect this interaction effect at an alpha of 0.05. Our target sample size was 40, however, our actual sample size of 37 fell just short, due to a covid lockdown-induced suspension of research operations. All participants gave informed consent and the Institutional Review Board of the University of California, Irvine, approved the study. All aspects of the study conformed to the guidelines of the 2013 WMA Declaration of Helsinki.

### Task and procedure

The study used a version of the social gambling task first introduced by Mistry & Liljeholm^11^, in which each of six numbered slots on a game board yielded a 10¢ reward with some probability. Participants were instructed at the beginning of the study that all monetary rewards were fictitious but should be treated as real. They were further told that, while they would receive initial training on the probabilities with which each slot yielded the 10¢ reward, they would not be told about any monetary outcomes during the actual gambling phase. They would, however, have access to decisions made by a group of “previous gamblers” given the same slot options, before making their choice on each gambling trial. The group of previous gamblers was stated to have been drawn from a cohort of students participating in the study during the previous academic quarter, and to have been labeled based on their performance in the task as high (H) vs. low (L) earners. Thus, participants made their gambling decisions given information about the norm judgment of their peers, the expertise of those peers, and previously acquired knowledge regarding the expected monetary value of each available option.

**Figure 1:**
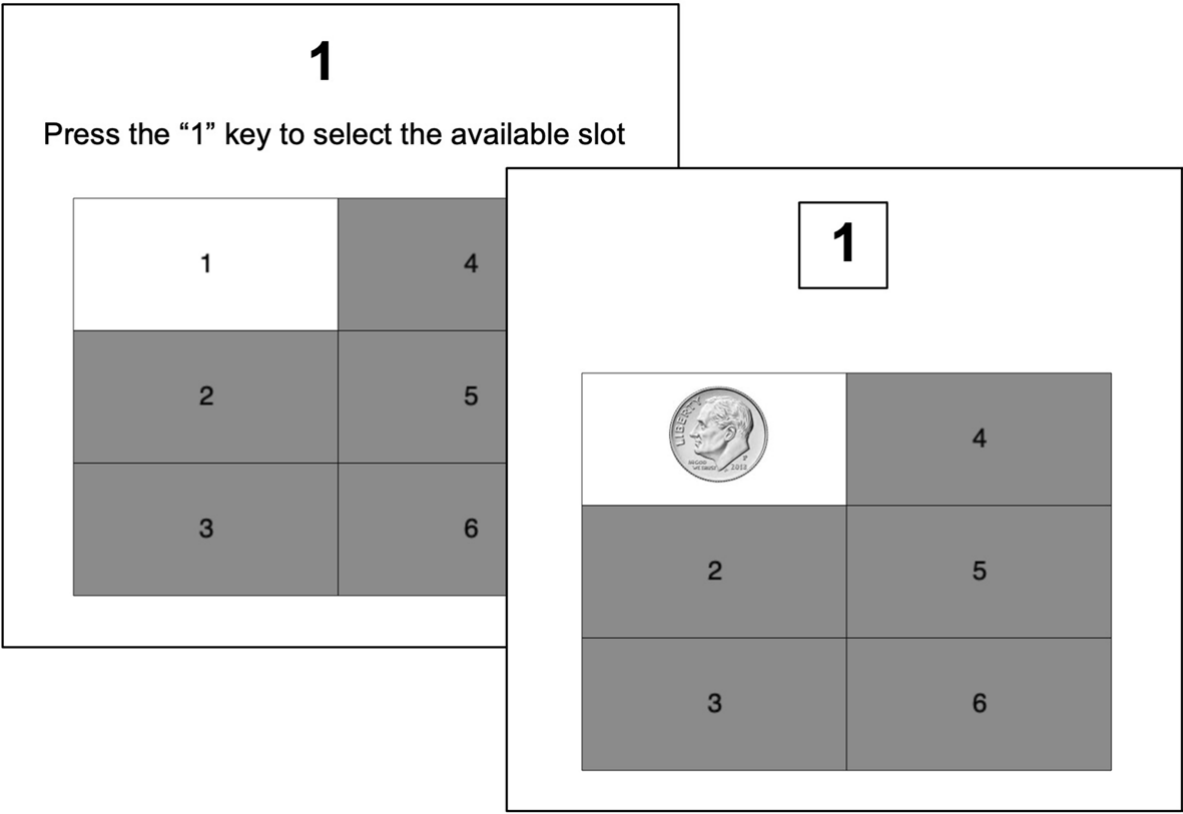
Training on slot reward probabilities. Choice and feedback screen on a trial in which slot 1 produced a 10-cent reward.

In the first phase, participants were trained on the reward probability of each slot on the game board until they were able to rate each slot within 0.2 of its programmed probability. To ensure equal sampling, each slot was highlighted on 10 consecutive trials indicating its availability. Once the participant selected the slot, an image of 10¢ or a red “X” graphic was superimposed on the slot (see Fig. 1), with relative frequencies corresponding to the probability with which the slot produced the 10¢ reward. Following 10 trials with a particular slot, the participant had to rate the reward probability for that slot within 0.2 of the programmed probability, or else the 10 trials were repeated. After receiving training on, and successfully rating, each slot option, participants were presented with each of the slots in random order, and again had to rate the reward probability of each slot within 0.2 of its programmed probability; if the rating of any one slot was not within 0.2 of its programmed probability, they were required to repeat the entire training phase. At the end of the experiment, to assess retention, participants again rated the probability of the 10¢ reward for each slot.

**Figure 2:**
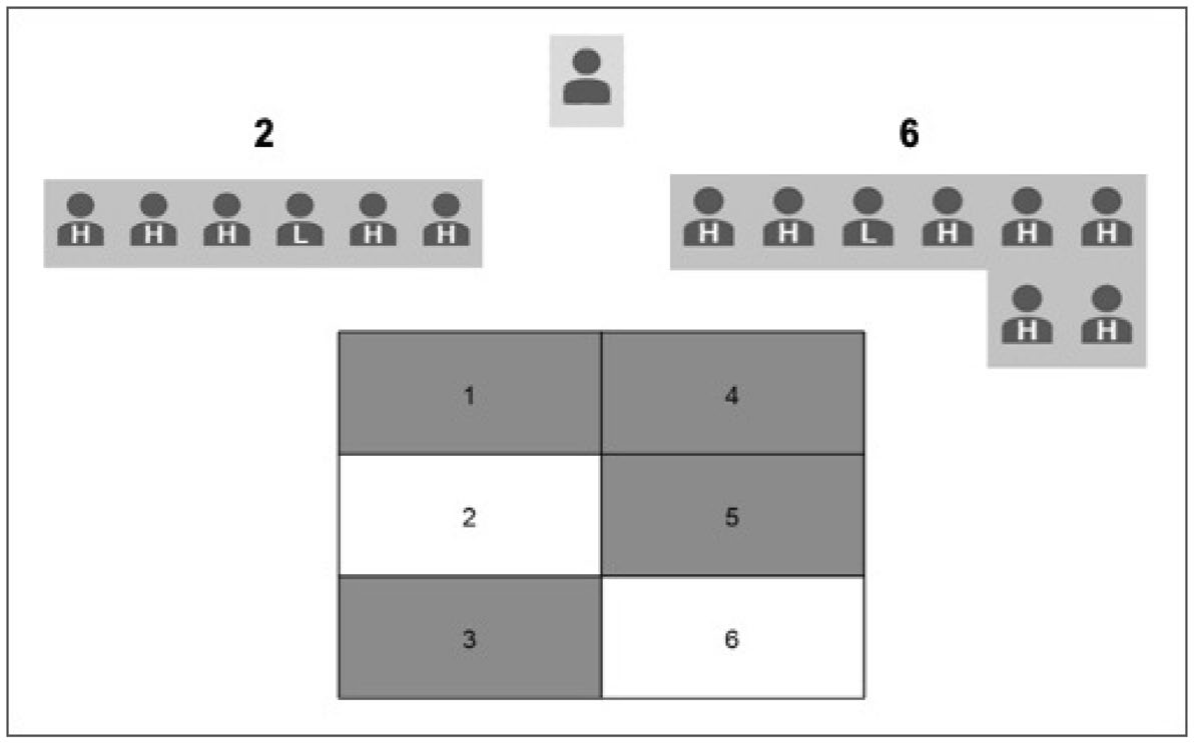
Choice screen on a trial in the social gambling phase. Participants use the left and right arrow keys to select slot 2 or 6, respectively. The participant is represented by the top middle avatar, and ostensible previous gamblers, labeled as either low earners (L) or high earners (H), are positioned under slot identifiers positioned at the top left and right of the screen. After making a selection, the participant’s avatar aligns with the previous players beneath the selected slot identifier.

On each trial in the subsequent gambling phase, illustrated in Fig. 2, participants chose between two available slot options, highlighted on the board and indicated by corresponding numbers printed on the top left and right sides of the screen. A panel of gray icons aligned beneath available options indicated the decisions of 16 ostensible previous gamblers given the same choice. Each previous gambler was labeled with an H or L, to indicate high and low previous earnings (henceforth expertise), respectively. Once a participant pressed a key to indicate selection of an available slot, the participant’s avatar, displayed at the top center of the screen at the onset of the trial, moved below the chosen option to align with the previous gamblers already displayed beneath that option. Participants were instructed that monetary earnings would not be revealed until the end of the experiment, but that they should assume that all monetary outcomes were consistent with the reward probabilities established in the initial training phase. This was done to avoid additional learning about monetary rewards during the gambling phase.

**Figure 3:**
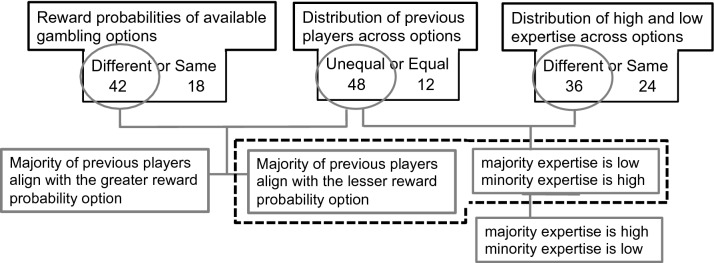
A graphical representation of the combination of variables of stakes, majority expertise and minority expertise into choice conditions, with one example condition shown encapsulated by the dotted boundary. All variables were manipulated within subject. Numbers indicate the total number of trials available at each level of each variable, from which subject-specific trials were subsequently drawn (see methods).

A flow chart of variables, levels, and their combination into 12 unique conditions is presented in Fig. 3. First, the two available gambling options on a given trial could have equal or different reward probabilities (two of the six game board slots had a 0.2 probability of reward, two had a 0.5 probability of reward, and two had a 0.8 probability of reward, yielding no difference, a small (0.3) difference, or a large (0.6) difference). Second, previous players could be equally or unequally distributed across the two gambling options—when unequal, the majority (a 0.63 to 0.75 proportion of the total number of 16 previous gamblers) could endorsed the option with a greater, lesser, or equal reward probability. Finally, the proportion of expertise, either high (0.83–1.0) or low (0.18–0.25), among previous gamblers could be the same or differ across gambling options. Differences within levels (e.g., a 0.3 vs. 0.6 difference in reward probabilities, or a 0.83 vs. 1.0 high proportions of expertise) were included to simulate plausible variance but were not distinguished in the grouping of levels into conditions, which only reflected categorical differences (e.g., different vs. identical reward probabilities or a high vs. low proportion of experts).

Specifically, these variables and their levels were combined, as illustrated in Fig. 3, into 14 unique within-subject conditions. In addition to those listed in Table 1, there were two conditions for which the reward probability differed across options, while previous players were equally split, and a high expertise proportion was associated with either the greater (6 trials) or lesser (6 trials) reward probability. The combination for which reward probabilities, numbers of previous gamblers, *and* proportions of expertise were all the same across options, yielding identical alternatives, was not included in the study. Note that the full design includes conditions in which the majority selected the gambling option with a greater reward probability—although these are less informative, since both payoffs and conformity indicate the same option, and therefore not part of our primary hypothesis, they were nevertheless included to assess the influence of minority expertise, as well as for plausibility (it would seem highly unlikely that the greater reward option was never selected by a majority of previous gamblers). To avoid easily discernible patterns reflecting experimental manipulations, natural variance was induced by randomly drawing the trials from the 12 conditions listed in Table 1, with the constraint that there must be at least 1 and at most 7 trials in each. As a results, while each participant received a total of 60 gambling trials, the relative number of trials from each condition differed across participants. The resulting mean number of trials in each condition, averaged across participants, is shown, with standard deviations, in the 5^th^ column of Table 1. The order of trials was randomized.

**Table 1:** Combination of reward and expertise variables into 12 within-subject conditions.

Reward probabilities	Majority choice	Majority expertise	Minority expertise	Mean # of trials $\pm$ SD
Different	Lesser	High	High	3.97 $\pm 1.9$
Different	Lesser	High	Low	3.62 $\pm 1.3$
Different	Lesser	Low	High	3.91 $\pm 1.5$
Different	Lesser	Low	Low	4.00 $\pm 1.6$
Different	Greater	High	High	3.83 $\pm 1.4$
Different	Greater	High	Low	4.1 $1\pm 1.8$
Different	Greater	Low	High	3.76 $\pm 1.5$
Different	Greater	Low	Low	3.65 $\pm 1.5$
Equal	–	High	High	3.62 $\pm 1.7$
Equal	–	High	Low	3.70 $\pm 1.5$
Equal	–	Low	High	3.76 $\pm 1.2$
Equal	–	Low	Low	3.78 $\pm 1.5$

The 2nd column indicates whether the majority of ostensible previous gamblers selected the option with a lesser or greater reward probability. Two additional conditions, for which ostensible previous gamblers where evenly divided across options (i.e., now majority) were included in the study but not listed in the table (see text).

The measure of primary interest was the proportion of trials on which participants choose the option endorsed by the majority of ostensible previous gamblers. We predicted that the influence of minority expertise would be particularly pronounced when a high-expertise majority endorsed the incorrect (lower reward) option—i.e., when majority expertise and objective reward probabilities were most clearly at odds. At the conclusion of the gambling phase, participants again rated the reward probability for each slot on the gambling board, presented once in random order—the accuracy (i.e., mean deviation from programmed probabilities) of these ratings for each participant was used as a measure of objective competency. Finally, at the end of the study, participants filled out the Rosenberg Self-Esteem Scale—a well-validated, 10-item, global self-worth assessment tool ranging from 0 (low self-esteem) to 40 (high self-esteem)^15^. We hypothesized that, when majority alignment came at a cost, objective competence and global self-esteem would both predict conformity, reflecting informational and normative incentives, respectively.

### Debriefing

Immediately upon completing the experiment, participants were informed that the decisions made by “previous gamblers” had in fact been generated by a computer algorithm. They were then given the option to withdraw their data from the study in light of this new information. All participants gave written consent to having their data included in the study after learning of the deception.

## Results

**Figure 4:**
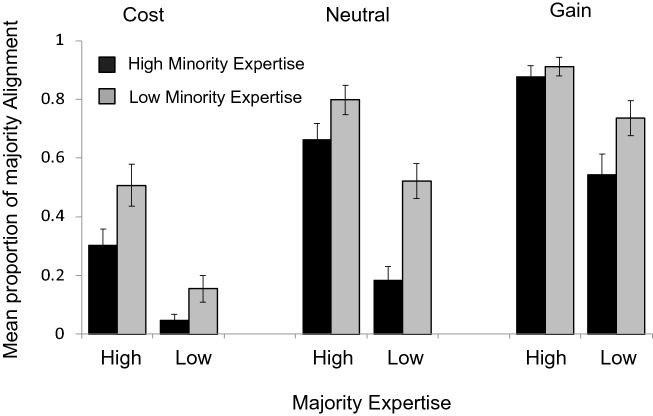
Results*.* Mean proportion of choosing the option endorsed by the majority of previous gamblers given that the majority endorsed an option with a lower (Cost), greater (Gain), or equal (Neutral) reward probability than the option endorsed by the minority, and given the High vs. Low earnings (i.e., expertise) of Majority and Minority members, respectively. Error bars = SEM.

Mean objective competence and self-esteem scores were 0.06 $\pm$ 0.10 and 29.02 $\pm$ 4.21, respectively. The proportion of trials on which participants conformed (i.e., chose the option endorsed by the majority of ostensible previous gamblers), across conditions defined by (1) whether the majority-endorsed option had a lower, equal or greater probability of a hypothetical monetary reward relative to the alternative option, (2) whether the expertise of the majority was high or low, and (3) whether the expertise of the minority was high or low, is plotted in Fig. 4. Note, again, that while our primary hypothesis concerns the influences of majority and minority expertise on conformity that comes at a cost relative to no cost, scenarios in which conformity led to a gain were included for completeness, in Fig. 4 as well as in the analyses detailed below.

A three-way repeated measures analysis of variance (ANOVA), with Stakes, Majority Expertise and Minority Expertise as within-subjects variables, was performed on the data. As predicted, there was a three-way interaction between Stakes, Majority Expertise and Minority Expertise, F(2,72) = 3.76, *p* = 0.03, ${\eta }_{p}^{2}$=0.10. Post hoc pairwise comparisons, using FDR correction, revealed that the difference between high and low minority expertise was significantly smaller when majority expertise was low than when it was high was in the Cost condition [t(36) = 2.39, adjusted *p*_*FDR*_ = 0.035)] while a non-significant (p = 0.18) trend in the *opposite* direction, such that the difference between high and low minority expertise was smaller when majority expertise was high than when it was low, was observed in the Neutral condition. A significant influence of minority expertise on conformity was found for all comparisons (all adjusted *p*_*FDR*_ < 0.05) except those in which the majority endorsed the gambling option with the greater reward probability *and* majority expertise was high (p = 0.382). The ANOVA also yielded significant two-way interactions between Stakes and Majority Expertise, (F(2,72) = 3.28, *p* = 0.043, ${\eta }_{p}^{2}$=0.08), Stakes and Minority Expertise, (F(2,72) = 3.82, *p* = 0.027, ${\eta }_{p}^{2}$=0.1), and Minority and Majority Expertise, (F(1,36) = 5.06, *p* = 0.031, ${\eta }_{p}^{2}$=0.12), as well as main effects of Stakes (F(2,72) = 48.09, *p* < 0.001, ${\eta }_{p}^{2}$=0.57), Majority expertise (F(1,36) = 44.211, *p* < 0.001, ${\eta }_{p}^{2}$=0.55), and Minority Expertise (F(1,36) = 32.11, *p* < 0.001, ${\eta }_{p}^{2}$=0.47).

Recall that the number of trials in each condition, generated by a combination of the levels of our independent variables, was drawn randomly from a uniform distribution with bounds 1 and 7, for each participant. The mean number of trials in each condition is listed, with standard deviations, in Table 1. To ensure that differences between conditions in the degree of conformity did not reflect the number of trials per condition, an ANOVA was performed on the number of trials, rather than the proportion of conforming decisions, in each condition, for each participant. There were no significant main effects or interactions involving the relative number of trials across conditions (all *p’s* >  = 0.15), ruling this factor out as the source of our effects.

Next, to assess whether the tendency to conform at a cost varied, across participants, with levels of objective competence (i.e., the accuracy of post-gambling reward probability ratings) and self-esteem, we computed the Pearson correlation coefficient between these variables and each of the four conditions in which the majority endorsed the gambling option with the lower reward probability. The only effects that survived FDR correction for multiple comparisons were the correlations between objective competency and conformity to a high-expertise but incorrect majority, whether countered by a high-expertise minority (*r* = − 0.56, adjusted *p*_*FDR*_ = 0.003) or a low expertise minority (*r* = − 0.44, adjusted *p*_*FDR*_ = 0.024). Specifically, the greater the objective competence, the less likely a participant was to conform to an incorrect majority made up primarily of experts.

In addition to the hypothesis-driven correlation analyses, we assessed the relationship between order of participation and conformity, for each of the 12 experimental conditions included in the ANOVA. This was done to gauge whether susceptibility to the deception decreased over time, given that participation in the study spanned a three-month period, providing ample time for subjects to disclose the deception to subsequent participants (despite instructions not to do so). While none of these correlations survived correction for multiple comparison, uncorrected p-values suggested that, when both gambling options had the same reward probabilities, and the minority expertise was high, a later order of participation increased conformity to a high-expertise majority (uncorrected p = 0.012, r = 0.41) but *decreased* conformity to a low-expertise majority (uncorrected p = 0.045, r = − 0.33). We consider it unlikely that these highly selective, and uncorrected, effects reflect a general decrease in the efficacy of the deception over time.

Finally, at a reviewer’s request, a post hoc power analysis was performed on the interaction score used for our a priori power calculation (i.e., the difference in conformity between high and low minority expertise, given high vs. low majority expertise, when conformity came at cost vs. when reward probabilities were equal). This contrast yielded an effect size of 0.42, with an observed power of 0.7 for our sample of 37 participants. Note that this post hoc estimate of power far exceed levels associated with sign (Type S) and magnitude (Type M) errors^16^. When combining our small pilot sample with our main sample, for a total sample size of 51, the interaction score yielded an effect size of 0.46, with an observed power of 0.9.

## Discussion

The increased tendency toward group alignment with increased group expertise is often attributed to an informational treatment of others’ decisions as proxies for decision outcomes. Here, using a simple gambling task with hypothetical monetary rewards, we assessed how the expertise of a group majority might interact with decision stakes, and with the expertise of a countering minority group. We found that the difference in conformity across high vs. low minority expertise was greater when majority expertise was low than when majority expertise was high, but only if conformity did *not* come at a cost. Specifically, when conformity with a low-expertise majority *did* come at a cost, it was extremely unlikely, regardless of the expertise of the countering minority. We also found that, when the decision to conform came at a cost and majority expertise was high, conformity was significantly predicted by lower objective competence, defined as accuracy of post-gambling reward probability ratings, regardless of the level of minority expertise.

An important caveat for interpreting these results is the fact that our sample was made up almost exclusively (~ 87%) of females. Although some evidence suggests that women show slightly greater levels of conformity than males (See^17^ for a meta-analysis), results on the role of gender in conformity are mixed. For example, using a perceptual judgment task in which subjects estimated the number of dots on the screen, with a high or low, male or female, expert confederate, Crano^7^ found no main effect of the gender of the participant, but did find that this variable interacted with the gender of the confederate (such that conformity was greater when the participant and confederate had the same gender). More recently, Wijenayake et al.^18^ probed the effects of gender on conformity using an online quiz, in which, for each multiple-choice question, participants were given the opportunity to revise their answer after seeing that they had selected an option chosen by either a majority or minority of ostensible previous, male or female, respondents. They found no significant gender difference in conformity but did find that both men and women typically conformed more to a majority with a large proportion of males for questions perceived as stereotypically male, and to a majority with a large proportion of females for questions perceived as stereotypically female, suggesting that gender biases reflect assumptions about expertise. Further research is needed to assess whether the interactions between stakes and majority and minority expertise, reported here, can be generalized across genders.

Other limitations also constrain the interpretation of our findings. Most notably, we used hypothetical monetary outcomes. While the use of fictitious money is prevalent throughout the decision sciences, with direct comparisons showing equivalence to real monetary rewards at both behavioral and neural levels^19–22^, and while our results clearly demonstrate the incentive of hypothetical rewards, with conformity dramatically increasing across a loss vs. gain of fictitious money, it is nonetheless important to confirm generalizability to real money in future studies. Another limitation is the use of fictitious, and graphically represented, ostensible others. Again, such methods are pervasive in research on both informational^12^ and normative^23,24^ conformity, but it is reasonable to assume that group pressures are diminished relative to real-world interactions. A final concern relates to the use of objective competence and the Rosenberg self-esteem scale as predictors of conformity, as these were likely insufficient to gauge confidence in oneself, and in one’s knowledge of reward probabilities. In particular, even those participants who demonstrated high accuracy in post-gambling probability ratings might have experienced high levels of uncertainty about objective reward probabilities when confronted with an inaccurate high-expertise majority. Future studies will be aimed at identifying transient changes in confidence about decision outcomes.

Several previous studies have shown that conformity increases with perceived group expertise^7,25,26^ and, further, that this influence is modulated by perceived self-competence^5,6^. Often, the interaction between self-competence and group expertise has been argued to reflect an informational basis for conformity—the less knowledgeable you are, and the more informed the apparent judgements of others, the more likely you are to treat those others’ judgments as objective evidence^12,18^. Many aspects of our results are consistent with this characterization of social influence. First, we found that majority alignment increased with majority expertise and decreased with the expertise of a countering minority. The significant influence of an expert vs. non-expert minority endorsing a different option than the majority is, to our knowledge, a novel finding. Second, objective competence—defined as the accuracy of post-gambling estimates of slot reward probabilities—significantly predicted majority alignment *only* when majority expertise was high, and conformity came at a cost. In other words, the poorer a participant’s knowledge of the objective reward probabilities, the more likely that participant was to align with an *incorrect* majority decision, but only if the majority was made up primarily of experts. The modulating influence of objective competence on deference to high, but not low, majority expertise is entirely consistent with an informational basis of conformity.

On the other hand, some of the obtained effects are difficult to reconcile with a purely informational basis for majority alignment. For example, while it is not surprising that conformity is reduced when it comes at an objective cost, it is not clear why, if conformity reflects the treatment of others’ decisions as evidence, such evidence should be weighed *less* heavily when conformity comes at a cost? Indeed, in order to selectively reject the advice of the majority when conforming comes at a cost, one must *know* that conformity comes at a cost, suggesting that the informational gain is redundant. Likewise, the reduced influence of minority expertise specifically when alignment with a low-expertise majority came at a cost suggests that conformity costs differentially modulate the perceived validity of evidence provided by a high- vs. low-expertise minority. Finally, recall that objective competence *only* predicted the degree of high-expertise majority alignment when such conformity came at a cost: This suggests that, when majority alignment is economically inconsequential, conformity may be primarily driven by the subjective value of belonging to a group. We suggest that this normative influence on conformity is in competition with informational factors, and thus overshadowed by salient differences in cost and expertise across options. Further work is needed to dissociate the relative contributions of decision cost, objective competence, and perceived majority and minority expertise to conformity in economic choice.

In summary, we used a gambling task to assess the influences of a dissenting minority, proportion of experts, and decision cost on the tendency to align with a majority decision. We found that the influence of minority expertise was greatest when majority expertise was low and reward probabilities were the same across gambling options. Moreover, participants’ knowledge of objective reward probabilities only predicted majority alignment when a high-expert majority endorsed the gambling option with the lower reward probability. While further research is needed to determine the generality of these findings, particularly with respect to the use of hypothetical money and graphically represented ostensible others, the results provide a novel contribution to a growing literature on cognitive and motivational determinants of social conformity.
